# Pre-operative CT-guided wire localization of a retroperitoneal mass for laparoscopic surgery

**DOI:** 10.1259/bjrcr.20150416

**Published:** 2016-07-27

**Authors:** Hsi-Ping Chi, Roberto Tozzi, Niall R Moore

**Affiliations:** ^1^Department of Radiology, Oxford University Hospitals NHS Foundation Trust, UK; ^2^University of Oxford, Oxford, UK; ^3^Nuffield Department of Obstetrics and Gynaecology, University of Oxford, Oxford, UK; ^4^Nuffield Department of Medicine, University of Oxford, Oxford, UK

## Abstract

A 48-year-old female with a 9-year history of granulosa cell tumour presented with progression of a mass in the left flank after the recent gradual rise of her inhibin B levels. She had experienced multiple recurrences and had undergone multiple operations to resect previous tumour recurrences. Initial laparoscopy did not identify the most recent recurrent mass. MRI was repeated a month after the surgery; it confirmed the presence of the mass and demonstrated an increase in the size of the tumour. Owing to difficulties in finding the tumour, a CT-guided wire localization of the mass was performed immediately prior to a second elective laparoscopy, leading to successful removal of the recurrent granulosa cell tumour. We describe the use of a conventional localization wire under CT guidance to facilitate the resection of a unique retroperitoneal tumour. This case report discusses the current applications of the wire localization technique, the evolution of the hook wire system, the potential complications that may occur and the factors influencing the likelihood of success of wire localization in the retroperitoneal space.

## Clinical presentation

In 2006, a 37-year-old female patient presented with acute onset abdominal pain. A CT study demonstrated a 17 cm left ovarian mass with features of a torted benign cystic teratoma. She underwent laparoscopic left oophorectomy. The pathology report confirmed a mature benign cystic teratoma of the ovary with areas of granulosa cell tumour but no signs of breach of the ovarian capsule. Staining for inhibin was weakly positive. Completion surgery of right salpingo-oophorectomy, subtotal hysterectomy and omentectomy was subsequently performed. Post-operative monitoring was performed by measurement of serum inhibin levels every 4 months and interval CT imaging. In late 2009, CT imaging detected three pelvic masses suggestive of disease recurrence. Histology of the masses resected during laparotomy in early 2010 showed recurrent granulosa cell tumour with high-grade sarcomatous elements.

Between 2010 and 2012, there were two more laparoscopic resections of recurrent masses. The patient was started on bleomycin, cisplatin and etoposide chemotherapy in May 2012 to control her recurrent ovarian disease with subsequent maintenance letrozole.

**Figure 1.**
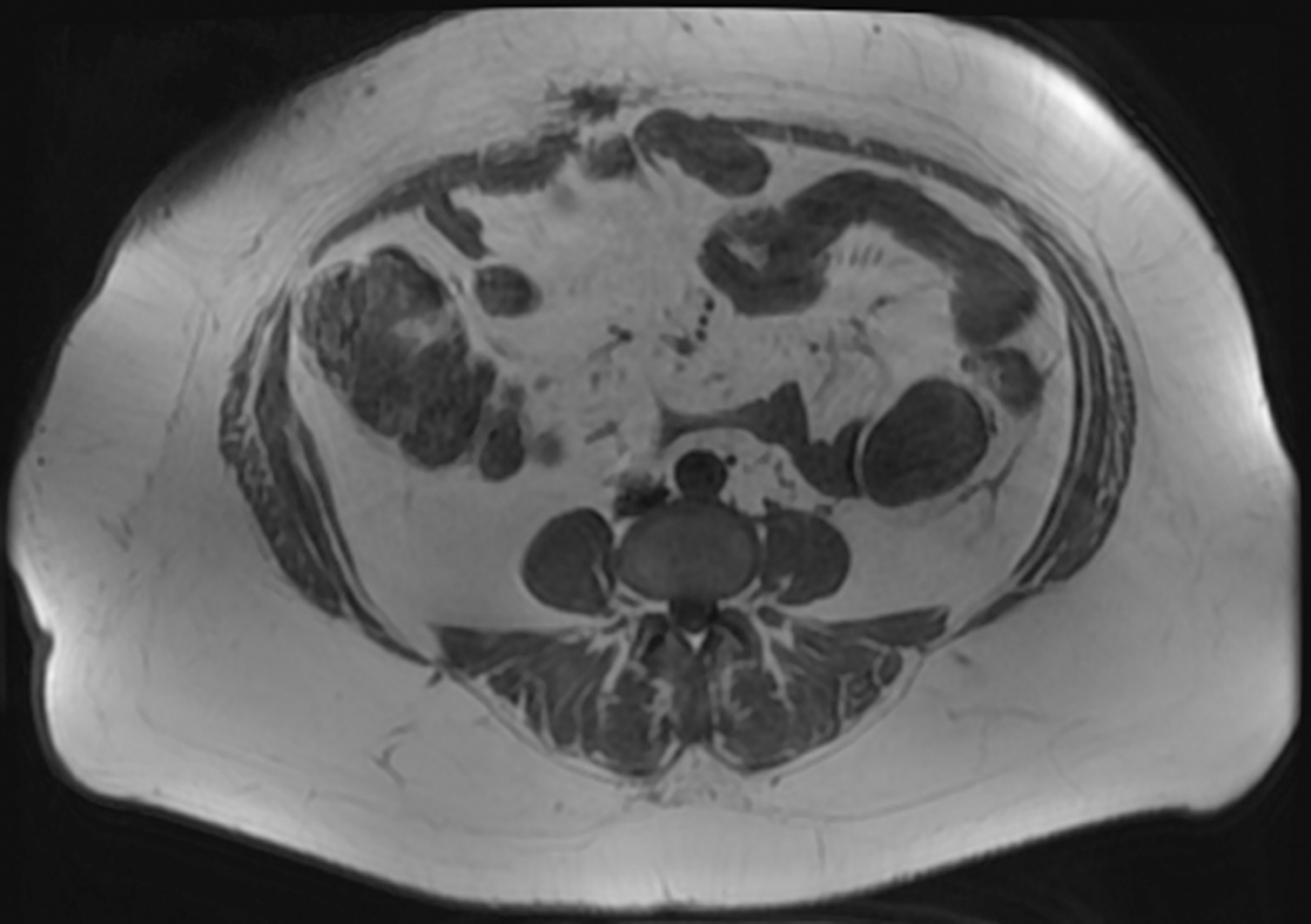
Axial *T*_2_ weighted image demonstrating the tumour posteromedial to the descending colon and lateral to the ileum.

In 2014, her inhibin B levels increased to 222 and MRI showed progression of nodules in the left flank and the right iliac fossa. In November 2014, she underwent an exploratory laparoscopic surgery with extensive adhesiolysis and bowel mobilization in an attempt to resect the nodules; there were no suspicious features and no tumour was found. At a subsequent multidisciplinary team meeting, a repeat MRI was planned in order to determine if the mass was still present. In December 2014, MRI confirmed the presence of the mass in the left flank and demonstrated an increase in its size (Figure 1).

Further multidisciplinary team discussion was in favour of excision of the mass but open laparotomy was not considered a practical option owing to patient size. Repeat laparoscopic excision was planned but additional pre-operative localization of the retroperitoneal mass was requested. The route for placement of the hook wire was discussed with the surgeon in order to facilitate subsequent laparoscopy. Supine positioning of the patient for laparoscopy meant that a posterior or posterolateral oblique approach for the hook wire was considered impractical. Accordingly, a lateral retrocolic route was chosen as the most appropriate approach.

## Differential diagnoses

The differential diagnosis includes recurrent granulosa cell tumour and another type of retroperitoneal tumour.

## Investigation/imaging findings

**Figure 2.**
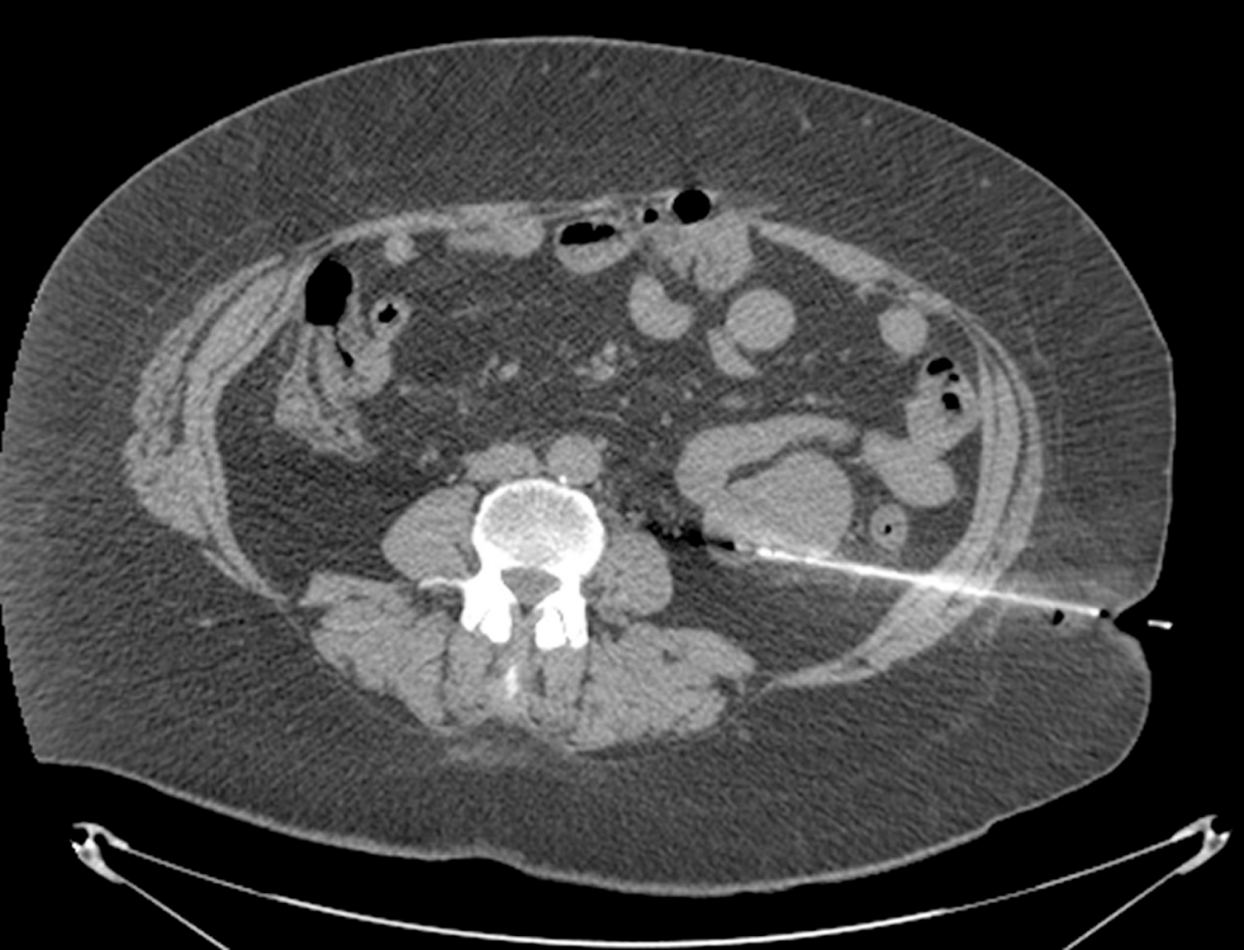
Positioning of the Hawkins hook wire needle *via* a lateral retrocolic approach into the posterior margin of the tumour.

**Figure 3.**
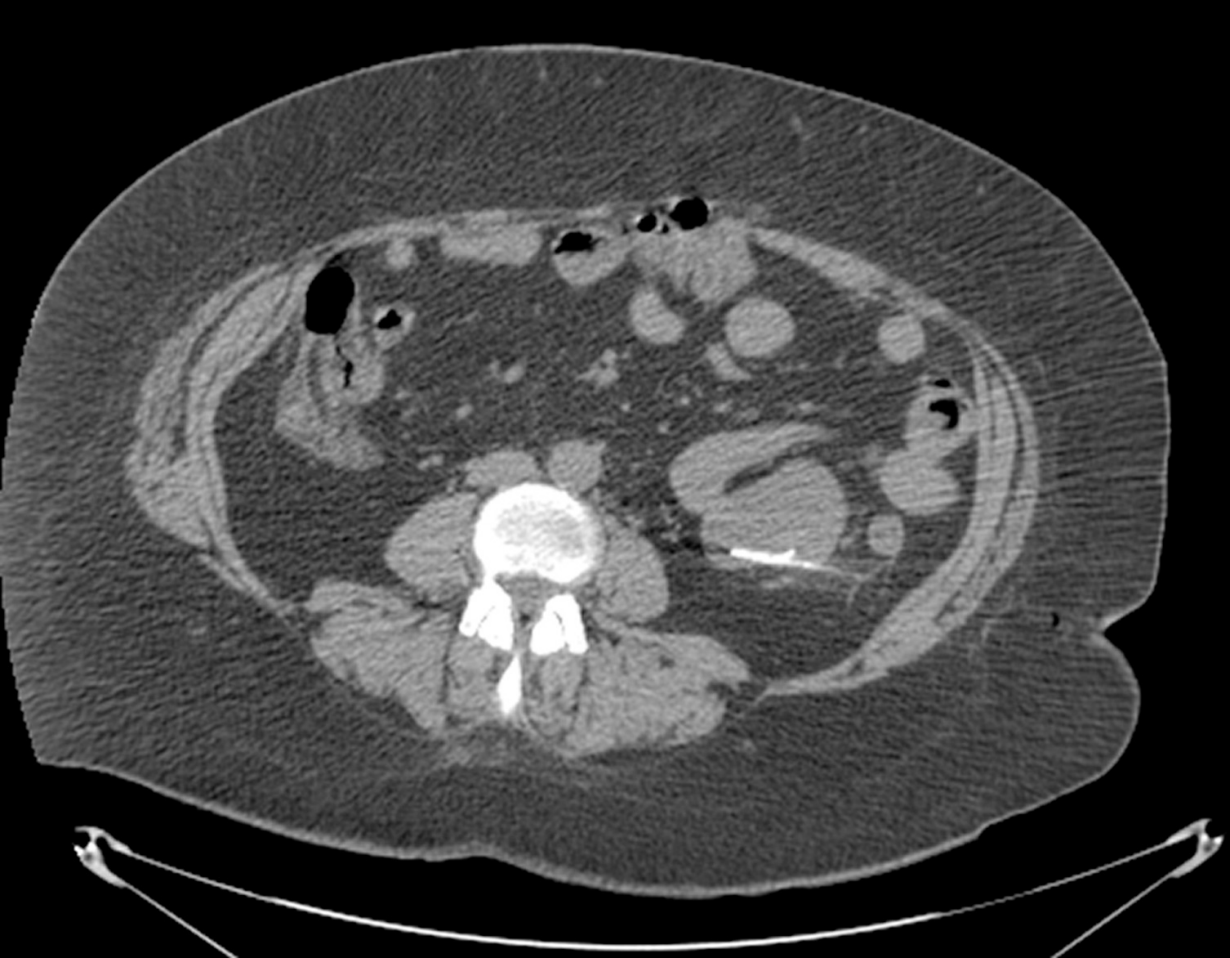
Hook wire deployed within the tumour.

In 2015, the patient was admitted to the radiology day case unit for CT-guided localization using a Hawkins I BLN (Angiotech, Gainsville, FL) breast tumour hook wire localization needle. After obtaining informed consent, the skin was sterilized with chlorhexidine gluconate 20% (Hydrex, Ecolab, Leeds, UK). The skin, subcutaneous tissues and the track to the tumour were anaesthetized by infiltration of 1% lidocaine hydrochloride (Hameln, Gloucester, UK). A 16-gauge needle (Quick-core, Cook Medical, Bloomington, IN) was used for infiltration and this was advanced to the posterior margin of the tumour under non-contrast CT guidance. Once the position of the mass was confirmed by a conventional short helical acquisition, the hook wire needle was advanced through the outer needle into the posterior layers of the mass (Figure 2). Once satisfactory positioning was confirmed, the hook wire was deployed to stabilize the needle within the mass (Figure 3). The wire external to the patient’s body was further secured by sterile tape to the skin before transferring the patient to the operating theatre.

## Treatment

**Figure 4.**
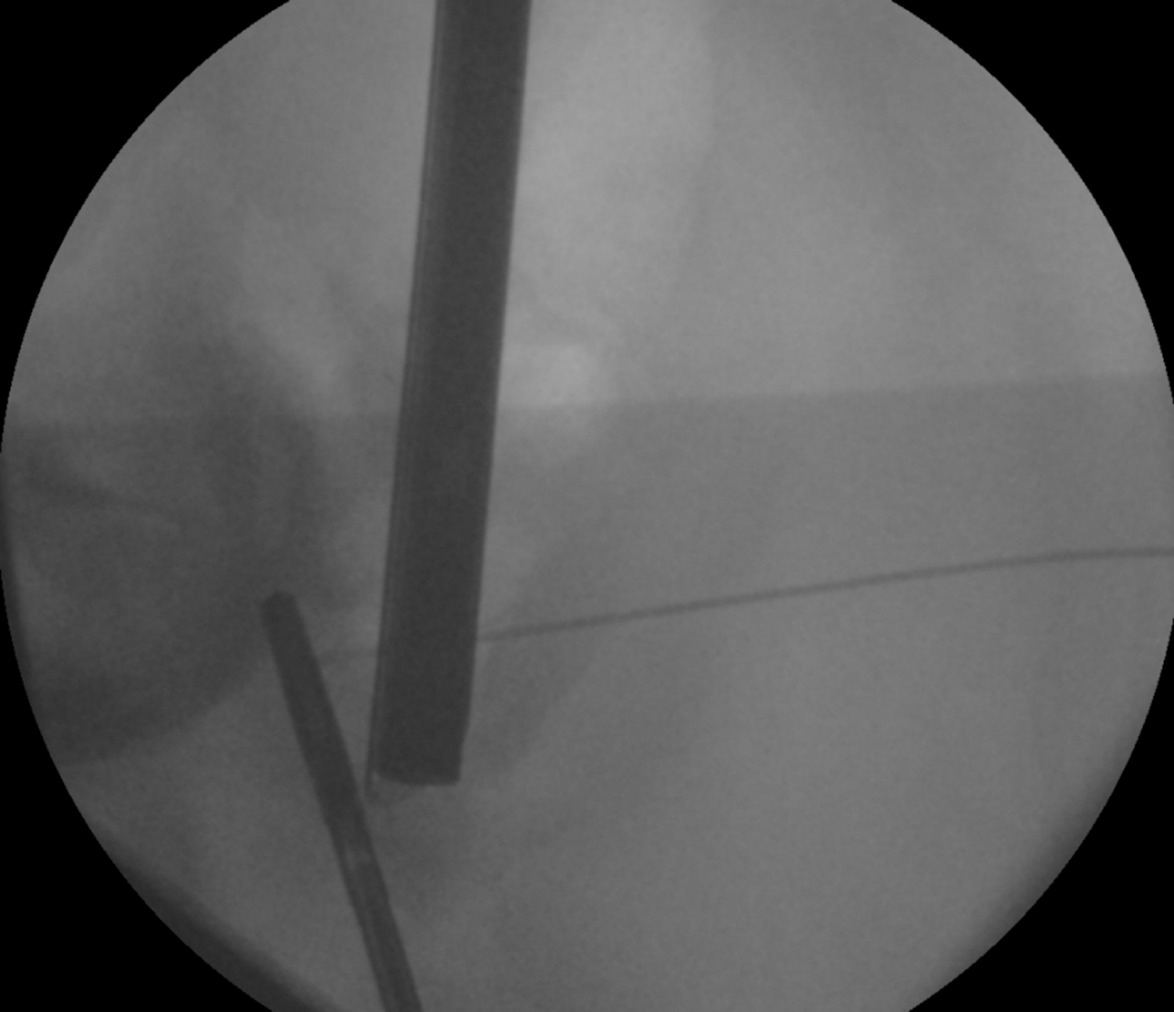
Intra-operative image intensifier image during laparoscopy demonstrating targeting of the laparoscope onto the hook wire.

The patient underwent laparoscopic surgery with a standard five-port transperitoneal technique commencing at the Palmer’s point, with the secondary ports being inserted under direct vision. After division of the adhesions, the hook wire was identified using a C-arm fluoroscopy machine (Figure 4). The mass was excised completely and extracted intact by the use of an endobag, avoiding spillage and contamination, through one of the port incisions and subsequently sent for histological analysis. This confirmed a 45 × 35 × 15 mm granulosa cell tumour, which contained mixed sarcomatoid, moiré silk and trabecular elements.

## Outcome

The guided laparoscopic surgery enabled successful removal of the recurrent granulosa cell tumour, and the patient made an uncomplicated post-operative recovery. The patient was discharged on the first post-operative day.

## Follow-up

At the oncology follow-up, there was no clinical evidence of relapse identified. The appearances of the nodule in the right iliac fossa remained stable at the 8-month radiological follow-up.

## Discussion

Granulosa cell tumours are tumours of the sex-cord stromal cells. They represent a rare subset of ovarian neoplasm with a comparatively indolent natural history. The diagnosis is most frequently made in middle-aged patients, although the incidence follows a bimodal distribution in age.^1^ They are commonly diagnosed at a relatively early stage and are often associated with late recurrences. Features, including stage, tumour size and mitotic index, have been associated with the likelihood of recurrence.^1^ The presence of residual disease and advanced patient age are potential additional adverse prognostic indicators.^1,2^ Patients may present with abdominal pain or menstrual disorders. Surgical treatment is influenced by the consideration of preserving reproductive function and the stage of the disease.

Insertion of a hook wire is a common technique used for localization of impalpable breast lesions before surgery. More recently, the technique has been used for localization of pulmonary nodules in video-assisted thoracoscopic surgery (VATS). It has also been used in several different settings under CT guidance, including localization of musculoskeletal lesions such as osteosarcoma, other lesions such as brachial plexus neurofibroma and metastatic melanoma in a lymph node.^3,4^ It has been used under ultrasound guidance for a number of applications, including testis-sparing surgery in the treatment of testicular cancer, pre-operative localization of neck lesions and pre-excision localization of intramuscular haemangioma.^5–7^

This is a case where hook wire localization has been used for localizing a retroperitoneal mass under CT guidance. The surgeon believes that the procedure would not have been possible without the wire. In fact, a previous attempt without the wire failed to identify the mass, hence the idea of wire placement. The wire was essential, as it clearly located the area where the lesion was. The difficulty in exposing the lesion was secondary to it being retrocolic and antimesenteric. The surgery consisted of full mobilization of the descending colon up to the splenic flexure and access to the space between the left kidney and the psoas muscle. Following full isolation of the left ureter and exposure of the common iliac and gonadal vessels, the wire was followed all the way into the lesion, which was then mobilized from the dorsal aspect of the descending colon and the left lateral aspect of the aorta and removed intact.

The original hook wire was used for localization of impalpable breast lesions. Based upon this established indication, different variations of the wire localization system have been developed. A recent report demonstrated the advantage of using a Kopans wire that has been modified by a thick reinforced segment to localize pulmonary ground-glass nodules using VATS.^8^ This study emphasized the fact that VATS can only be offered if the surgeon can visualize or palpate the target nodule, which can be better achieved by using the modified Kopans wire, as the reinforced segment was designed to be positioned within the lesion to aid palpation. In our case, a standard Hawkins localization needle was used.

There are a number of complications for using the hook wire system to localize small lesions. When using the hook wire to localize a retroperitoneal mass, the potential risks include risks common to all invasive procedures, such as infection and bleeding; risks specific to wire localization technique, such as wire dislodgement; risks specific to the anatomical site, such as injury to the bowel or any other intra-abdominal organs; and risks specific to the indicated procedure, such as seeding of malignant cells of the target tumour.

It is not feasible to quantify the risk of seeding precisely, as the needle track was not excised and the risks of seeding are unknown in the reported case. However, it is the breach of the tumour capsule and shedding of cells along the biopsy track resulting from the biopsy procedure that is associated with the risks of seeding. The delivery needle was positioned adjacent to but not breaching the tumour capsule, hence negating the chance of tumour seeding along the track. The purpose of the needle was to deliver the localization wire and allow the wire to advance into the mass. No biopsy samples were obtained and the needle was removed after the hook wire was placed in the desired position. The wire did not go through the track of entry again because the hook wire was removed together with the mass in an antegrade fashion at the time of surgery, that is, internally *via* the laparoscope and not externally through the skin. As a result, the surrounding tissue did not come in contact with the segment of the wire that was inserted into the mass. This removal technique minimized the chance of seeding by the hook wire.

The operation was scheduled immediately after the radiological procedure such that there was minimal time for wire dislocation to occur. In addition, minimal wire displacement was ensured by both internal and external measures. Internal stability was ensured by the hook on the wire at the end of the needle, which engaged into the tumour tissue akin to a fish hook. External stability was provided by the firm taping of the external part of the wire to the skin to prevent dislodgement, as described above.

There are a number of factors that determine the success rate of localization by the hook wire system. In VATS, these include patient age, solidity of the nodule, location of the nodule, and the distance between the nodule and the pleural surface.^9^ The authors of this study showed that localization of nodules in the lower zones of the lungs was related to higher failure rates, and suggested that this may be explained by the wider excursion of the lower zones of the lungs during respiratory movements. In the same study, it was shown that sufficient distance between the nodule and the pleural surface was related to higher success rates, probably owing to better anchoring of the hook wire in the target mass. When localizing a retroperitoneal mass, it may be logical to follow the same principles and hypothesize that sufficient distance between the target mass and the abdominal wall would be a factor that would influence the likely success of localization. Similarly, movement resulting from breathing or bowel peristalsis may make accurate needle positioning difficult.

## Conclusions

The hook wire localization technique, traditionally used in localizing impalpable breast lesions prior to breast surgery and localizing pulmonary nodules prior to VATS has been used in localizing a granulosa cell tumour in the retroperitoneal space. This facilitated the surgery after an initial exploratory surgery failed to identify the mass. In the reported case, the patient had extensive adhesions in the abdomen owing to multiple recurrences of granulosa cell tumour, requiring multiple laparotomies and laparoscopies, and the application of this established technique allowed the surgeon to identify the mass intra-operatively and remove it successfully.

## Learning points

1. The hook wire localization technique is widely used to localize impalpable breast lesions and small pulmonary nodules, but can also be used for the localization of masses in the retroperitoneal space.
2. In the case discussed, no complications were identified. However, there are a number of potential complications, including infection, bleeding and haematoma formation, injury to bowel or other intra-abdominal organs, and wire dislodgement.
3. A number of factors may influence the likelihood of success of wire localization in the retroperitoneal space. These include the architecture and consistency of the target mass, the distance between the target and the abdominal wall and the amount of movement owing to breathing and bowel peristalsis.
4. Additional experience with the technique will help in ascertaining the efficacy and complication of wire localization in the retroperitoneal space; it may help in identifying the optimal type of wire to be used for this application and may help in elucidating the factors influencing the success rate of this localization technique.

## Consent

The patient has given her informed consent in writing for the publication of her case and the associated images and data.
